# What can we learn from the five-year African swine fever epidemic in Asia?

**DOI:** 10.3389/fvets.2023.1273417

**Published:** 2023-09-28

**Authors:** Satoshi Ito, Nijiho Kawaguchi, Jaime Bosch, Cecilia Aguilar-Vega, Jose Manuel Sánchez-Vizcaíno

**Affiliations:** ^1^VISAVET Health Surveillance Center, Complutense University of Madrid, Madrid, Spain; ^2^Department of Animal Health, Faculty of Veterinary Medicine, Complutense University of Madrid, Madrid, Spain; ^3^Division of Molecular Pathobiology, Hokkaido University International Institute for Zoonosis Control, Sapporo, Japan

**Keywords:** African swine fever, Asia, domestic pig, wild boar, infectious disease, epidemiology, disease control, risk assessment

## Abstract

Today’s global swine industry is exposed to the unprecedented threat of African swine fever (ASF). Asia, the site of the most recent epidemics, could serve as a huge viral reservoir for the rest of the world given the severity of the damage, the huge swine industry, and the high volume of trade with other countries around the world. As the majority of ASF notifications in Asia today originate from pig farms, the movement of live pigs and associated pork products are considered critical control points for disease management. Particularly, small-scale or backyard farms with low biosecurity levels are considered major risk factors. Meanwhile, wild boars account for most notified cases in some countries and regions, which makes the epidemiological scenario different from that in other Asian countries. As such, the current epidemic situation and higher risk factors differ widely between these countries. A variety of studies on ASF control have been conducted and many valuable insights have been obtained in Asia; nevertheless, the overall picture of the epidemic is still unclear. The purpose of this review is to provide an accurate picture of the epidemic situation across Asia, focusing on each subregion to comprehensively explain the disease outbreak. The knowledge gained from the ASF epidemics experienced in Asia over the past 5 years would be useful for disease control in areas that are already infected, such as Europe, as well as for non-affected areas to address preventive measures. To this end, the review includes two aspects: a descriptive analytical review based on publicly available databases showing overall epidemic trends, and an individualized review at the subregional level based on the available literature.

## 1. Introduction

African Swine Fever (ASF), caused by the ASF virus (ASFV), is a contagious disease of domestic and wild pigs (1) and is one of the most influential transboundary animal diseases for the livestock industry in the world today. The clinical stages can be divided into four main categories: peracute, acute, subacute, and chronic (2); however, symptoms vary according to the balance between the virulence of the virus strain and host immunity, contributing to the variety of regional epidemiological scenarios. An essential aspect of this virus is its high environmental resistance, being well known for its ability to remain infectious for long periods under various conditions (3). Susceptible animals can be infected through direct contact with infected animals or indirect contact with contaminated materials (4, 5).

Asia is one of the main epidemic areas in the current global ASF epidemic; it accounts for more than half of the world’s pork production and plays an important role in world trade. Rather than controlling ASF, the epidemic situation is becoming more complex, raising fears that ASF could spread further around the world, primarily by the movement of contaminated materials. Given the history of multiple ASF jumps from Africa to Europe, it is possible that Asia could play a similar role in the near future. Asia has the potential to become the global reservoir of the virus due to its high pig farming densities and greater human and material traffic. This would pose further threats to Europe, one of the current major epidemic areas, and likewise to ASF-free countries or those in the process of eradication.

ASF was originally confined to Africa but has been spreading globally since its reintroduction into Europe in 2007. Within the same year of its entry into Georgia, outbreaks were reported in Armenia, Azerbaijan, and Russia. In 2014, the disease reached the European Union via Lithuania, Poland, Latvia, and Estonia. By the end of June 2023, ASF was confirmed in 23 European countries (6), posing a major threat to Western European countries with large pig farming populations, such as France and Spain. In Asia, ASF was first confirmed in China in 2018. Shortly afterward, a series of infections were reported in neighboring countries, and, to date, 18 countries and regions have reported ASF. In 2020, the first ASF outbreak in the Oceania region was reported in Papua New Guinea. The following year, 2021, ASF was confirmed in Haiti and the Dominican Republic, two Caribbean countries located in the middle of the North and South American continents, for the first time in about 40 years. As these epidemics demonstrate, ASF is now a global problem.

Relevant research is being conducted in Asia in a variety of fields, ranging from molecular biology to epidemiology, as well as economics. However, much remains unknown compared with Europe, where the ASF epidemic occurred earlier and several valuable studies have been carried out. Unique sociocultural and traditional practices may contribute to the maintenance and expansion of the disease, making it challenging to obtain a complete picture of the epidemic. ASF spread in Asia has been exceptionally rapid compared with Europe, where a total of 23 countries were infected in the 16 years since 2007, whereas only four countries were infected within the first 5 years. What lies behind such a rapid and extensive spread of the disease over a 5 years period? What are the differences or similarities with the epidemic in Europe, where the spread has been relatively slow compared with Asia? The answers to these questions will provide valuable information, not only for both regions but also for countries at risk of infection in the future.

This review collected nearly 5 years of information available regarding the ASF epidemic in Asia (August 1, 2018, to June 30, 2023) and summarized the epidemic status as well as relevant background knowledge across Asia. For this purpose, it includes two aspects: a descriptive analytical review based on publicly available databases to elucidate overall epidemic trends; and a literature-based individualistic review of each region. Quantitative epidemiological ASF data were obtained from the databases of two international organizations: the EMPRES Global Animal Disease Information System (EMPRES-i) of the Food and Agriculture Organization (FAO) of the United Nations (7) and the World Animal Health Information System (WAHIS) from the World Organisation for Animal Health (WOAH founded as OIE) (6). The EMPRES-i database contains information such as the date of observation, country, subregion, and geographic coordinates of where the event occurred. This database was used to elucidate the number of notifications in each country and their spatial distribution. In addition to the WOAH data, the database also includes information provided independently by each country’s institution, providing a more detailed notification count. The WAHIS database contains detailed epidemiological information, including the number of susceptible animals, the number of cases, the number of animals killed, and the epidemiological unit to which animals belong. This database was used to provide the first ASF event records and the number of infected or susceptible animals in each country. Scientific articles written in English from the beginning of 2017 to the end of June 2023 were reviewed in the PubMed database to obtain insights related to the epidemiological context. Relevant country data were retrieved from national databases or reliable online media as needed.

## 2. Overview of the ASF temporal trend in Asia

### 2.1. Introduction of ASF to Asia

In early March 2017, an ASF outbreak was reported on one backyard farm in the Irkutsk region of the Russian Federation, near the border with Mongolia. Since then, subsequent ASF outbreaks have occurred in Siberia and near the border with China, raising concerns about the disease entering Asian countries (8). Around spring of 2018, animals showing clinical signs similar to ASF began to be discovered in northeastern China (9–11) and, on August 3, 2018, ASF was officially reported in the northeastern Chinese city of Shenyang (10). The results of the phylogenetic tree based on partial sequences of the *p72* gene showed that the outbreak strain ASFV-SY18 isolated in China had a 100% nucleotide identity with the strains isolated in Georgia, Russia, and Estonia (Georgia 2007/1, Krasnodar 2012, Irkutsk 2017, and Estonia 2014), suggesting that the outbreak was caused by a pan-Russian ASFV strain (10). Several sources have been suspected for the initial introduction of ASFV into Asia, however, this remains unknown (12, 13).

### 2.2. ASF epidemic in Asia 2018–2023

**Figure 1:**
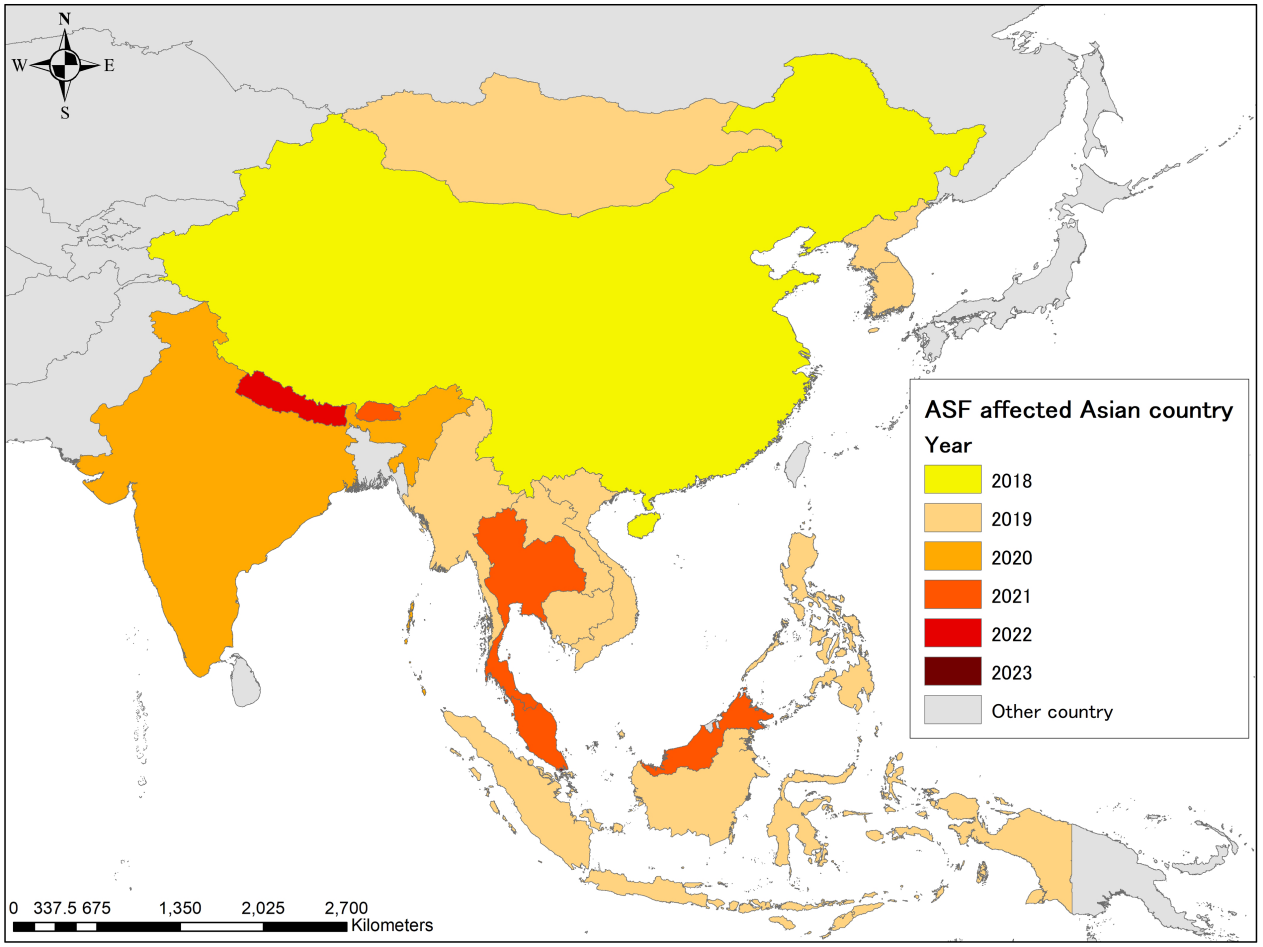
Year of the first confirmed African swine fever (ASF) case in infected Asian countries as of 30 June 2023. The map was depicted using ArcGIS 10.8.1 (ESRI, Redlands, CA, United States).

**Figure 2:**
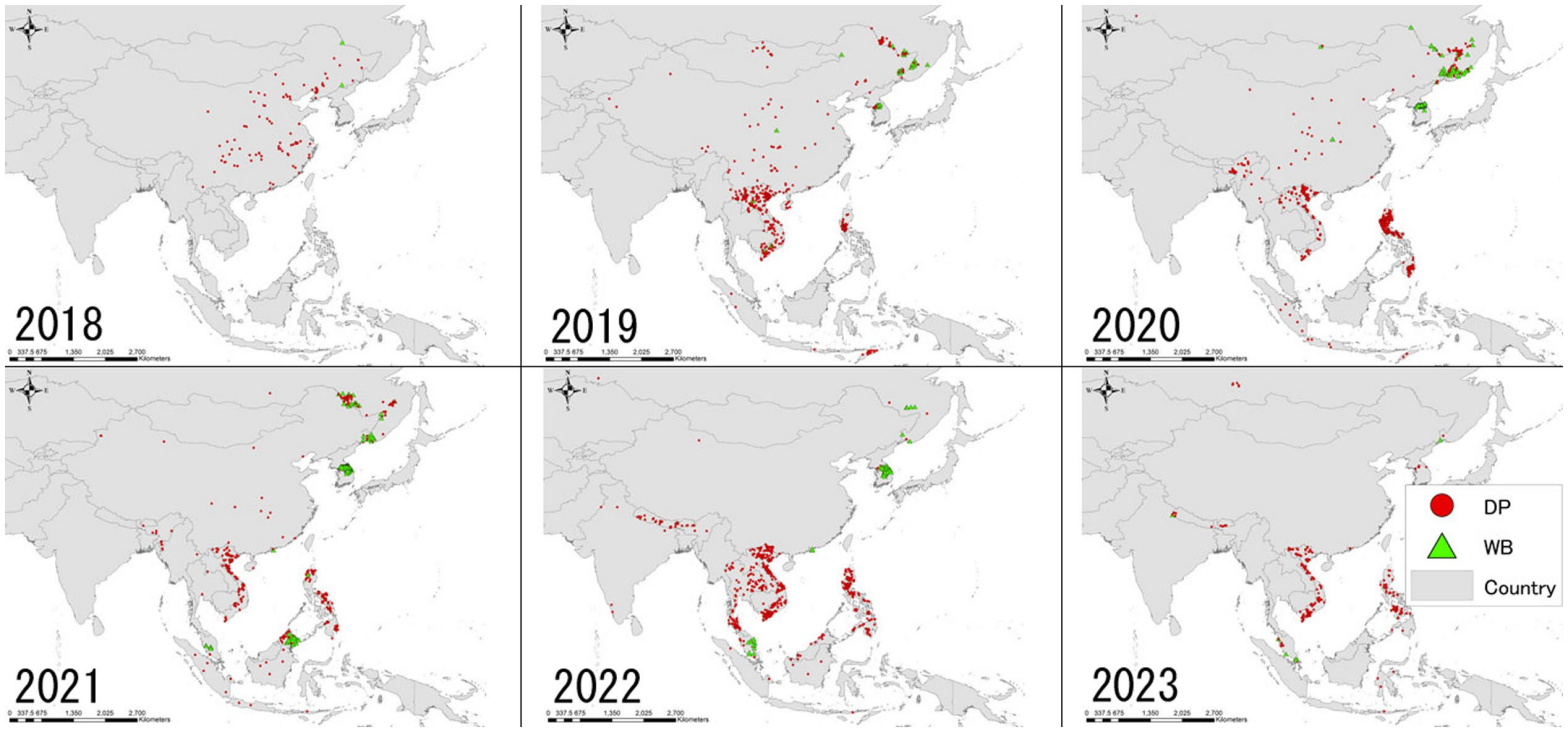
Annual trend of ASF spatial distribution in Asia including the geographically close Russian Far Eastern region as of June 30, 2023, based on the FAO EMPRES-i database. The map was depicted using ArcGIS 10.8.1 (ESRI, Redlands, CA, United States).

**Figure 3:**
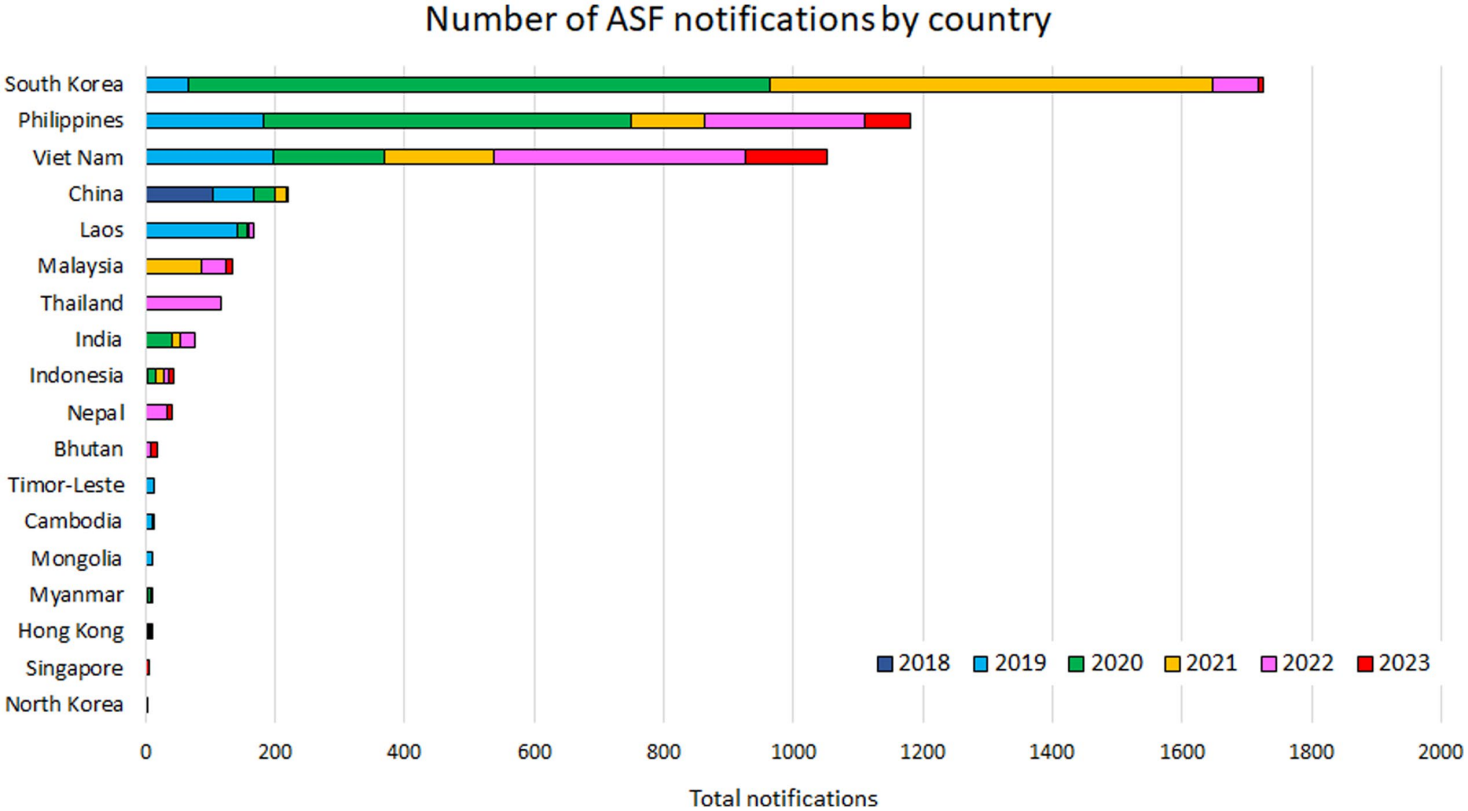
The number of annual and total ASF notifications by country as of June 30, 2023, based on the EMPRES-i database.

Based on the EMPRES-i database, China was the only Asian country infected with ASF in 2018, with a total of 104 outbreaks reported; the WAHIS database documented approximately at least 358,000 susceptible and 12,700 infected animals (Tables 1, 2). In 2019, the disease rapidly spread to neighboring East and Southeast Asia, reaching 11 countries and regions (Mongolia, Vietnam, Cambodia, Hong Kong, North Korea, Laos, the Philippines, Myanmar, Indonesia, Timor-Leste, and South Korea) (Figure 1) (6). A total of 695 notifications were recorded in the database that year, the majority originating from domestic pigs as well as a small number of wild boar cases (Table 2) (7). The rough distribution of ASF occurrences in 2020 was similar to that of 2019 (Figure 2), with the highest-ever number of notifications reported (1,743) due to the constant regional disease expansion in East and Southeast Asia (7). About half of these reports originated from wild boars, mainly because of the spread of ASF infection in wild boars in South Korea (7) (Table 3 and Figure 3). In the same year, India confirmed its first ASF outbreak in South Asia (6).

**Table 1:** Timeline of the first ASF notifications in affected Asian countries in domestic and wild suids.

Country	Domestic pig	Wild boar
China	2018/8	2018/11
Mongolia	2019/1	
Vietnam	2019/2	2019/5
Cambodia	2019/3	
North Korea	2019/5	
Hong Kong	2019/5	2021/9
Laos	2019/6	2019/8
Philippines	2019/7	2021/5
Myanmar	2019/8	
Indonesia	2019/9	
South Korea	2019/9	2019/10
Timor-Leste	2019/9	
India	2020/1	
Malaysia	2021/2	2021/2
Bhutan	2021/5	
Thailand	2021/11	
Nepal	2022/3	2023/3
Singapore	2023/4	2023/2

**Table 2:** Annual notifications of ASF in Asia.

	^a^Total notifications	^a^DP outbreak	^a^WB case	Susceptible animals	Infected animals
2018	104	102 (98.1%)	2 (1.9%)	358,309	12,700
2019	695	636 (91.1%)	59 (8.9%)	8,489,292	155,754
2020	1743	846 (48.5%)	897 (51.5%)	2,988,452	83,950
2021	1,105	389 (35.2%)	716 (64.8%)	70,617	9,980
2022	941	860 (91.4%)	81 (8.6%)	95,988	22,324
2023	248	241 (97.2%)	7 (2.8%)	375,751	37,171

DP, Domestic pig; WB, Wild boar.

a Total notifications are retrieved from the EMPRES-i database and susceptible/Infected animals are based on the WAHIS database.

**Table 3:** Total number of ASF notifications per host and country.

Country	DP outbreak	WB case	Total
South Korea	36 (2.1%)	1,690 (97.9%)	1726
Philippines	1,181 (99.9%)	1 (0.1%)	1,182
Vietnam	1,050 (99.7%)	3 (0.3%)	1,053
China	212 (97.2%)	6 (2.8%)	218
Laos	165 (98.8%)	2 (1.2%)	167
Malaysia	83 (61.9%)	51 (38.1%)	134
Thailand	118 (100%)	0 (0%)	118
India	76 (100%)	0 (0%)	76
Indonesia	43 (100%)	0 (0%)	43
Nepal	39 (97.5%)	1 (2.5%)	40
Bhutan	18 (100%)	0 (0%)	18
Timor-Leste	13 (100%)	0 (0%)	13
Cambodia	12 (100%)	0 (0%)	12
Mongolia	11 (100%)	0 (0%)	11
Myanmar	10 (100%)	0 (0%)	10
Hong Kong	5 (55.6%)	4 (44.4%)	9
Singapore	1 (20%)	4 (80%)	5
North Korea	1 (100%)	0 (0%)	1

^*^Notifications are based on the EMPRES-i database.

The overall distribution of ASF notifications was fairly similar to that of 2019 (Figure 2), however, around 65% of the notifications in 2021 involved wild boars in South Korea (Table 3 and Figure 3). New ASF outbreaks were confirmed in Malaysia, Bhutan, and Thailand in that year, of which some wild boar cases were reported in Malaysia (Figure 2). In addition to the continuous ASF spread throughout East and Southeast Asia, a certain number of ASF events in both domestic pigs and wild boars were consistently reported in the Russia Far East, along the border with China, between 2019 and 2021 (Figure 2). While ASF was newly confirmed in Nepal in 2022, official outbreak reports from China have declined significantly, with reports concentrated in Southeast Asian countries, particularly Thailand, Vietnam, and the Philippines (Figure 2). In February 2023, Singapore newly reported ASF infection in wild boars, bringing the total number of ASF-infected countries/regions in Asia to 18 (Table 1).

**Figure 4:**
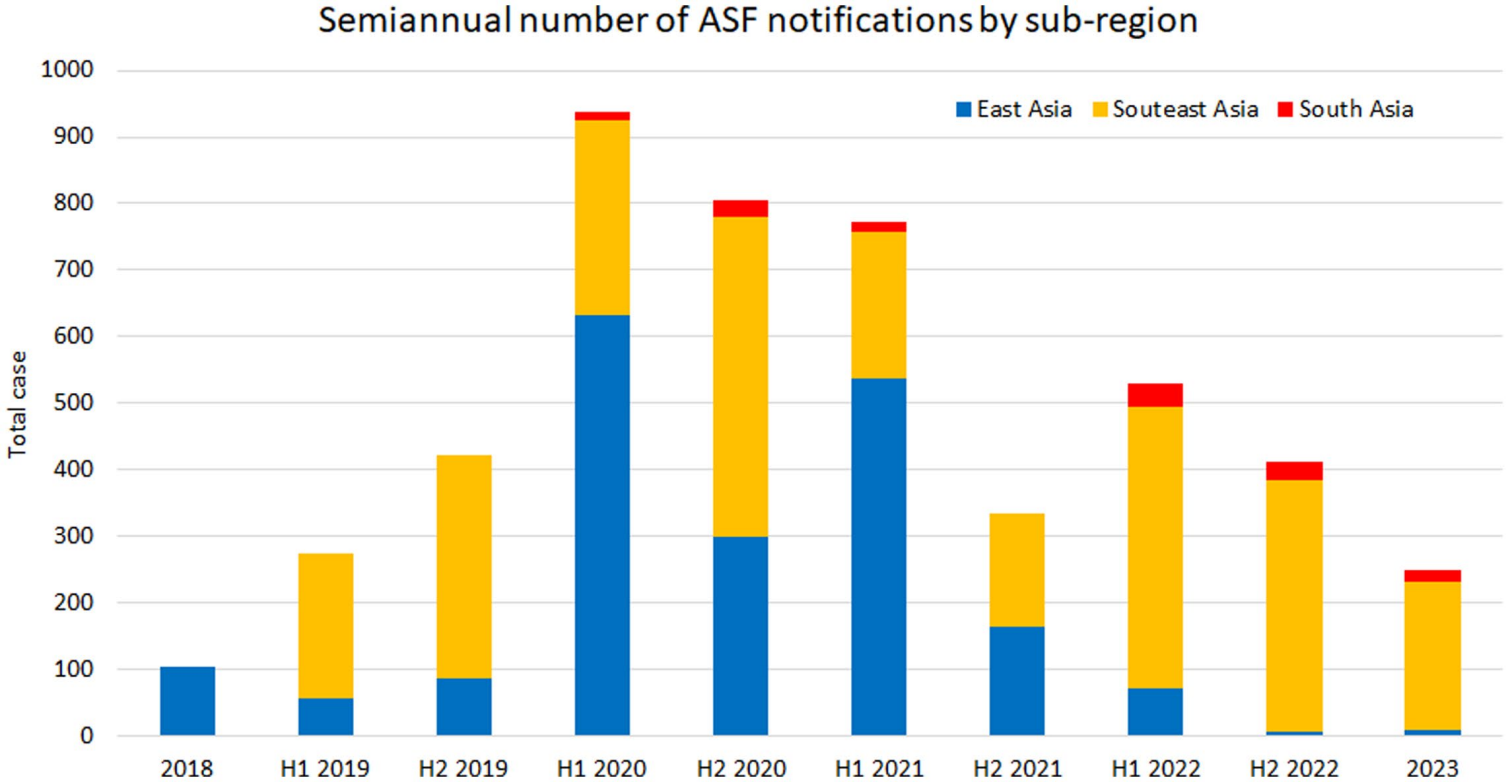
Number of semi-annual ASF notifications by subregion as of the end of June 2023, based on the FAO EMPRES-i database. H1 and H2 denote the first and second-half periods, respectively.

East Asia played a significant role in disease spread during the early stages of the ASF epidemic, primarily due to a nationwide outbreak in China. Subsequently, South Korean wild boar cases have accounted for most of the notifications in this subregion. On the other hand, a certain number of notifications have been continuously recorded in Southeast Asia since 2019 due to the widespread dissemination of the disease. South Asia has also continuously reported ASF notifications since 2020, with fewer than in other subregions (Figure 4). The number of notifications peaked in 2020 in the FAO EMPRES-i database; but ASF infections have been reported constantly from most of the affected countries as of the end of June 2023, suggesting that ASF is becoming endemic in Asia. At this point, a total of 4,836 notifications were recorded in the EMPRES-i database, of which 3,074 were domestic pig-related outbreaks and 1762 were wild boar cases.

The general epidemic trend over the past 5 years is that outbreaks associated with domestic pigs are observed throughout Asia, whereas wild boar cases are found mainly in certain countries/regions (Table 3). All ASF-infected countries have confirmed outbreaks in the domestic pig sector, while wild boar cases have been officially reported in 9 of the 18 infected countries/regions (Table 1). Different major transmission mechanisms have been reported in the early and late stages of epidemics in Asia. The spread of ASFV at the beginning of the outbreak, primarily in China, most likely occurred via the transportation of infected livestock, products, or fomites. In contrast, proximity to swine enterprises and direct contact may have contributed to the later stages of the epidemic in Southeast Asia (14).

## 3. ASF subregional update in East Asia

### 3.1. ASF epidemic status

**Figure 5:**
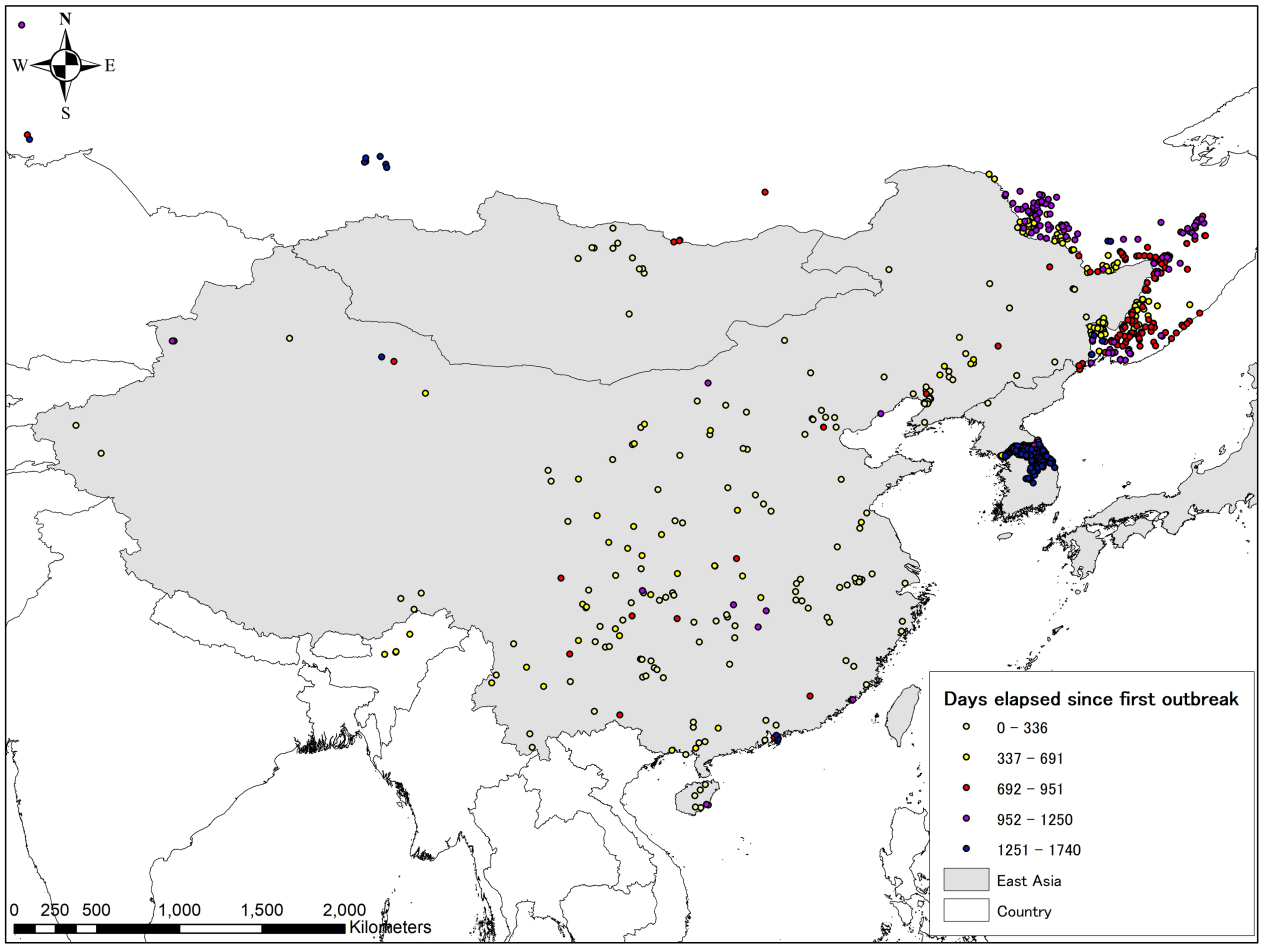
ASF evolution in Eastern Asia (including the Russian Far East) from August 1, 2018, to June 30, 2023, based on the EMPRES-i database. Lighter colors indicate earlier stages, darker colors indicate more recent occurrences. The map was depicted using ArcGIS 10.8.1 (ESRI, Redlands, CA, United States).

China, Hong Kong, Mongolia, South Korea, and North Korea are the ASF-infected countries/regions belonging to East Asia (Figure 5). After the rapid and widespread expansion of the disease in the early epidemic stages in China, official notifications are now sporadically reported from the entire country, thus becoming an endemic situation (15). Outbreaks have been observed in vast areas, many of which geographically overlap with large pig farming areas (16). There is a clear seasonal trend in the outbreaks, with the highest frequency of reports occurring during winter and spring. This is presumably due to a surge in consumer demand for pork during the Chinese New Year, one of China’s traditional festivals (17, 18). Many of these notifications are related to domestic pigs (97.2%), however, a certain number of ASF-positive wild boar cases are also reported (2.8%). For example, Hong Kong has recorded nine notifications in the EMPRES-i database since 2019, four of which are wild boar cases. ASF notification in both domestic and wild suids has also been confirmed in neighboring border areas, particularly in the Russian Far Eastern side of the border with China (19).

The phylogenetic analysis of ASFV isolated in China in 2018 showed great similarity to the highly virulent ASFV isolates from Eastern Europe (20). Likewise, recent ASFV isolates from East Asian countries such as Mongolia and South Korea were shown to be highly virulent genotype II ASF viruses with high homology to each other (10, 21–23). Due to this background, the highly virulent genotype II ASFV is generally considered to be predominant in this region (24) but this may not be true for China. Recent reports indicate that ASFV genotypes I and II, including lower virulent and recombinant strains, are simultaneously prevalent in Chinese swine herds, demonstrating that many different, genetically diverse ASFV strains are present (25–28). The non-hemadsorbing lower virulent genotype II and genotype I ASFVs have been repeatedly isolated in several Chinese provinces, which potentially relates to the production of illegal vaccines (29–31).

According to the EMPRES-i database, South Korea recorded the highest number of notifications in Asia as of the end of June 2023 (Figure 3). This is because the finding of one ASF-positive wild boar is frequently counted as one case, and the majority of notifications are reported in wild boars in South Korea. The number of official notifications peaked in 2020 and has been decreasing since then, however, the disease has not stopped spreading. Sporadic outbreaks have been reported on farms, spatially overlapping with the expansion of wild boar cases. Wild boars clearly play a pivotal role in the spread of ASF in South Korea. The current epidemic is most likely the result of multiple localized disease entries (32) or continuous transmission pressure along the border (33). In the early stages of the epidemic, a series of outbreaks were reported in neighboring areas following the initial ASF confirmation on a northwestern pig farm. Shortly thereafter, the first case in wild boars was officially confirmed (7), and, to date, numerous notifications, mainly from wild boar populations, have been reported. The infected areas continue to expand from the north to the south, serving as a corridor with the Taebaek Mountains, running north–south along the country’s eastern coast, and have reached the central part of the country at this point (33–35). These infected areas are considered suitable for wild boars but harder to access for surveillance and, thus, the actual epidemic status may not be properly understood (33). There are several theories as to the detailed mechanism, however, seasonality in the number of ASF notifications has been observed, with more notifications in winter and a minimum in summer, and outbreaks on pig farms are most common in autumn (36, 37).

Mongolia was the second Asian country to be infected with ASF, which was confirmed in a pig farm on January 10, 2019. Within 1 month of the initial outbreak, the disease affected 83 pig farms in seven provinces in the country, killing approximately 2,860 animals, representing about 10% of the total pig population (38). However, there have been no new outbreaks since early February 2019, and an end to the disease was declared on April 11 of the same year (38). North Korea reported one ASF outbreak in May 2019, after which no additional information is available. Japan and Taiwan are the only countries/regions in East Asia where ASF infections have not been reported as yet. In Japan, ASF-contaminated pork products are frequently detected at international ports, and the risk of ASF entry is estimated to be high (39). Similarly, quantitative risk assessment studies conducted in Taiwan have shown a very high risk of ASFV introduction (40). A dead pig that washed ashore in the territory was recorded as an ASF-positive individual in the EMPRES-i database (7, 40).

### 3.2. Pig industry and wild boar distribution

#### 3.2.1. Pig industry

East Asia is a region with a large pork industry, with China accounting for half of the world’s pork production (452.6 million heads as of April 2023) and South Korea being the world’s ninth-largest pork producer (41).

The rise in pork consumption driven by rapid economic development has led to an increase in the number of people seeking business opportunities in China. As a result, a complex, large trade network and value chain involving many different stakeholders has formed within the Chinese pork food system (42). The large-scale pig farming regions are mostly located in coastal areas and are divided into northern and southern regions. Pig farming is in the transition stage from bulk culture to large-scale agriculture, with 26 million households engaged in pig farming (15). The proportion of large-scale pig farming is increasing, nevertheless, the majority of farmers are small-scale for solely private consumption, where farms with less than 500 pigs account for about 99.4% of all pig farmers (15). After the ASF outbreak, guidelines for the prevention and control of ASF were issued to promote large-scale pig farming and reduce the number of small-scale farmers (43). Consequently, the number of small farmers may have decreased significantly but this pig production model is still likely to last a long time (15).

The swine industry is an indispensable part of South Korean agriculture, accounting for 30% of the livestock sector and producing more than 1 million tons of pork annually. As it is preferred over beef and chicken, pork is consumed in large quantities, thus it is also imported into the country (44). The highly intensive industry, with about 11.2 million heads divided among approximately 5,700 farms, is distributed mainly in the mid-western region of South Korea (45). The overall trend in the swine industry is toward structuring, modernization, and efficiency, with traditional small farms being closed, and larger, more modern swine farms on the rise (44). Recently, eight major on-farm quarantine facilities were established to improve the quarantine level for pig farms nationwide. These standards include the installation of internal and external fences with height criteria, the set-up of equipment essential for the disinfection and prevention of cross-contamination, and the use of nets to prevent the entry of wild animals and the storage of carcasses. The costs of installing these facilities are subsidized through the support program (46).

#### 3.2.2. Wild boar distribution

The Chinese wild boar population, including both wild and domesticated animals, is assumed to be very large and widely distributed throughout China. In addition, free-ranging feral pigs are present in many areas (47). The spatial density distribution of wild boars is unknown but it is estimated to be 2–5 heads/km^2^ in densely populated areas, with the total number reaching several million (48–50). Although reported wild boar cases are scarce, these conditions raise the possibility of their contribution to the maintenance of ASFV (15).

Before ASF introduction, the wild boar population in South Korea was growing rapidly, with an estimated population of 300,000 animals in a wide range of habitats, from forests to urban environments (32, 51, 52). Approximately 70% of the country is covered by forests and mountains, providing the optimal habitat for wild boars. Geographically, the Taebaek Mountains run north-south along the east coast of the Korean Peninsula, with two mountain ranges extending west in the central region and south-southwest in the central and southern regions, serving as a home for wild boars (53). On the border with North Korea, there is a 248 km-long and 4 km-wide barrier called the demilitarized zone (DMZ) that extends from the east to the west coast. Following this zone is a restricted civilian entry zone with a 7–15 km perimeter, which serves as a paradise for wildlife to thrive due to restricted human access (54). The average density of wild boars nationwide was reported by the government to be 4.1 heads/km^2^ as of October 2020 (55, 56). However, there are large regional differences, and it was noted that the density calculated after culling and searching for carcasses was approximately 10 heads/km^2^, indicating the possible underestimation of the population density (32).

### 3.3. Risk factors and control measures

The risk factors and countermeasures for ASF epidemics differ considerably depending on the importance of the activities associated with pigs and the role played by wild boars. In China, pig density is considered the most important risk factor, the various reasons for which are explained below. Long-distance transportation of pork and pigs was traditionally common due to the uneven distribution of pig farming industries. Measures restricting transportation to contain disease spread resulted in soaring pig prices and an increase in illegal transportation, leading to further long-distance transmission of ASFV (15). Given these considerations, the government implemented measures such as the registration and notification of pig transport vehicles, inspection of transport links as well as the detection of slaughter links (57). Furthermore, the country has been divided into five regions to restrict movement. Each region has an ASF-free zone, and only pigs from the free zones, breeding pigs, and piglets are allowed to move beyond their respective regions (58).

Distribution of contaminated pork and food waste is considered the main cause of outbreaks on small farms, while mechanical transmission of the virus by vehicles and personnel seems to be the main contributor to outbreaks on larger farms. Inadequate disinfection facilities and improper operation of cleaning and sterilization systems in slaughterhouses have been linked to several outbreaks, with a survey in 2019 reporting that, in some cities, 5% of slaughterhouses were contaminated with ASFV (59). For this purpose, the government announced a survey on the detection of ASFV in pig slaughtering and pork products distributed in January 2019 (60).

ASFV transmission via feeding leftover food to healthy pigs is known to be an important mode of viral spread (61) and is recognized as a major contributor during the early stage of the epidemic in China (62). As such, the government prohibited the feeding of food residues to pigs as of late 2018 (15, 63). These aforementioned risk factors were also raised in a previous systematic review of risk factors for ASF spread in China (64).

Wet markets play an important role in the sale of fresh meat (65, 66) and, therefore, a significant vulnerability of the pork food system in terms of managing the risk of ASFV transmission (67). Moreover, complex and large swine and pork production systems make it difficult to implement the “stamping out” tactics of complete destocking of contaminated facilities and tracing, as well as inspection of contacts (68). Possible animal disease control and prevention are influenced by those heavily involved in the value chain (traders, processors, retailers) rather than by farmers, thus complicating the implementation of ASF control measures. This makes ASF control in China more challenging compared with Europe and the current African pork food system (67).

To our knowledge, findings of ASFV in wild boars are very limited in China (69–71). Little importance has been placed on the role of wild boars in the ASF epidemic, however, it may be highly underestimated (64, 72). Despite the high density of wild boar populations and their large home range, the lack of information on their movements makes it difficult to assess the current situation (18, 70, 72). No ASF outbreaks involving tick infections have been reported in China as yet, however, more than 100 species of ticks are widespread throughout the country. While the role and mechanism of ticks in ASF transmission in China remain unknown, they have been identified as an important risk factor in various studies (15, 16, 73). Large knowledge gaps remain regarding the role of wild boars and ticks in ASFV transmission, thus underlining the need for further research (18, 64).

South Korea is considered to have implemented a relatively high level of control policy with a low ASF incidence on pig farms among Asian countries (36, 46). Contaminated vehicles and the movement of infected wild boars likely contributed to the ASFV transmission to pig farms; in particular, vehicle movement played a major role in the series of early outbreaks on farms (74). As soon as ASF is confirmed on a farm, movement restrictions and thorough disinfection are implemented for a certain period of time based on three levels of zoning (control zone, protection zone, and surveillance zone within a radius of 500 m, 3 km, and 3–10 km, respectively) (75). Persistent ASFV circulation in wild boars can be a continuous risk for pig farms. The accumulation of infected carcasses in the environment increases the risk of infectious agents flowing into farms in the summer due to natural disasters such as heavy rains and typhoons. In spring and fall, farm inspections and disinfection are intensified because of the increased risk of spatial contact with wild boars owing to increased farm work and mountain hikers, as well as the breeding season (76).

Disease containment measures among wild boars mainly consist of fencing, population control, and carcass removal. The fencing was installed in multiple stages, the first and second consisted of an electric fence enclosure of 1 to 2 km around the case report site and a semi-rigid wire mesh 1.5 m high placed approximately 5 to 10 km around it. A third fence was deployed across the country from west to east in areas 20 to 30 km away from the second fence to prevent further southward movement. Each time ASF cases were reported beyond the third fence, authorities enclosed the newly infected area (36, 75). The effectiveness of fences in preventing the spread of disease in wildlife is controversial (77), however, its role in South Korea is emphasized as a temporary measure to slow the transmission rate (78). Government-led search teams, organized nationwide at a regional scale, are constantly searching for wild boars, mainly around the infected areas (32, 79). The search was further prompted by offering a bounty for the discovery of the animal but this may have resulted in anthropogenic jumps in ASF spread. Persons without adequate biosecurity knowledge could have served as carriers of the virus by traversing infected areas during hunting and search operations (32, 34).

The Taebaek Mountains are an important pathway for the spread of ASF infection in South Korea. The high elevation of the mountains complicates consistent surveillance activities, thus making it challenging to precisely understand disease prevalence. Undetected infected carcasses may increase the concentration of virus in the environment and sustain the ASFV transmission cycle (33, 80). Recently, governments have focused on improving surveillance bias by introducing detection dogs and drones (81, 82).

In addition to the current virus strains in circulation, new ASFV introductions from abroad remain a major threat. The only land border with North Korea is fenced, so interactions are very limited. Accordingly, the quarantine framework is primarily based on border control as in island countries. While previous studies derived that the ASFV-introduction risk associated with the legal importation of live pigs and/or pork products is low (83), there are concerns regarding the risk of human-mediated pathways, such as illegal pork importation (36, 84). On the other hand, a study analyzing the distribution of ASF cases at the beginning of the epidemic identified proximity to North Korea as an important continuing risk factor (33). While wild boars are unlikely to pass through the border fence, the multiple rivers that span both countries will allow for the arrival of wild animal carcasses or portions thereof (33). The role of wildlife as vectors in the transmission dynamics of ASF in South Korea remains to be elucidated. In addition to wild boars, mammals such as raccoons, cats, and rodents, as well as birds, including vultures, are suggested to be possible spreaders of ASFV (85). In contrast, others believe that their role is limited and therefore controversial (32, 86), thus further research is required.

## 4. ASF subregional update in Southeast Asia

### 4.1. ASF epidemic status

**Figure 6:**
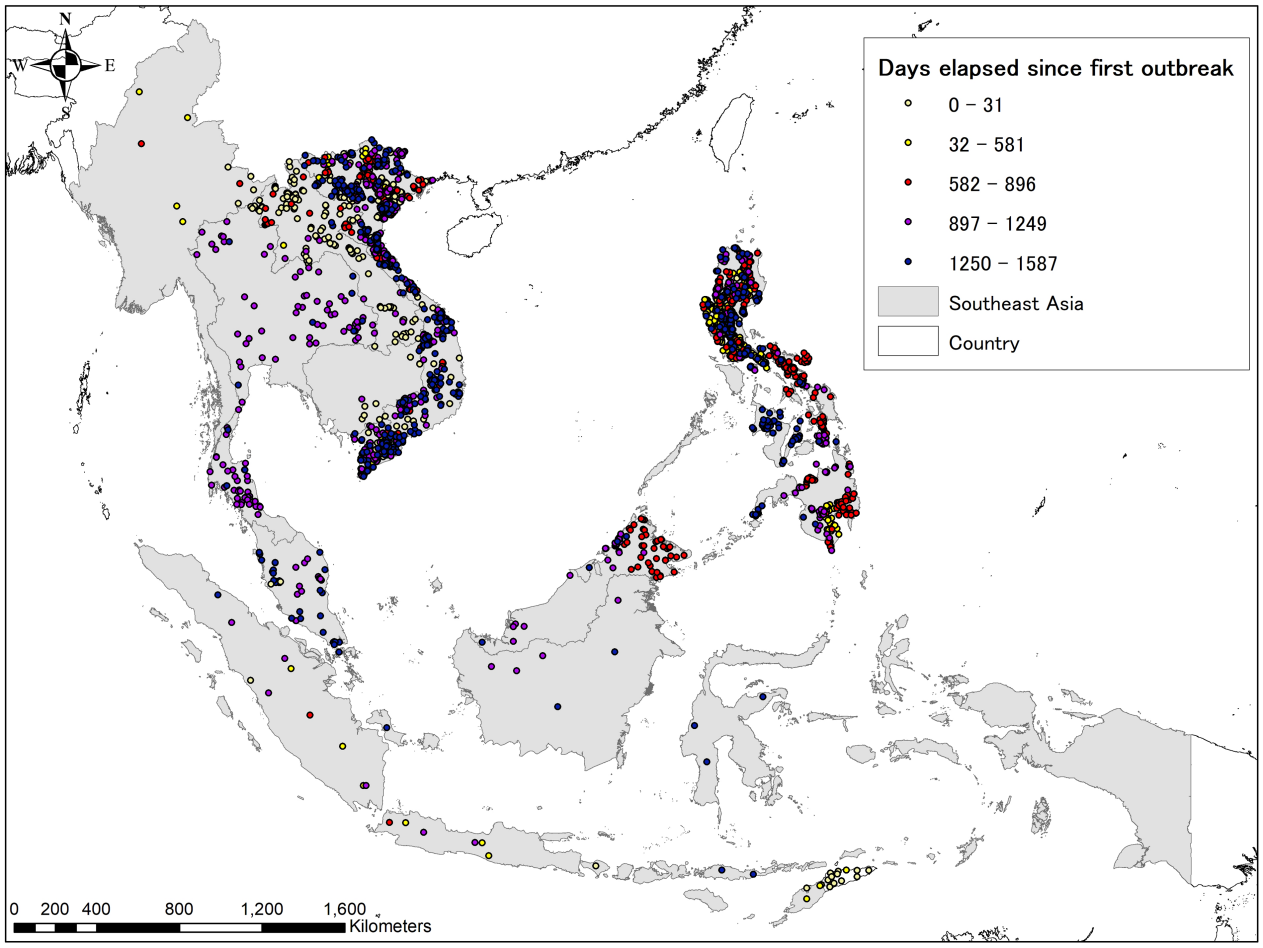
ASF evolution in Southeast Asia as of June 30, 2023, based on the EMPRES-i database. Lighter colors indicate earlier stages, darker colors indicate more recent occurrences in this region. The map was depicted using ArcGIS 10.8.1 (ESRI, Redlands, CA, United States).

ASF has been observed repeatedly in a wide area of Southeast Asia, with outbreaks confirmed in 10 countries (Vietnam, Cambodia, Laos, the Philippines, Myanmar, Indonesia, Timor-Leste, Malaysia, Thailand, and Singapore) (Figure 6). The first ASF notification in Southeast Asia was officially reported in February 2019, in Hung Yen province in northern Vietnam, and within the same year, six countries in the region confirmed ASF (Cambodia, Laos, the Philippines, Myanmar, Indonesia, and Timor-Leste). In that year, outbreaks were concentrated in Vietnam, Laos, and the Philippines, resulting in approximately 550 notifications in the EMPRES-i database. However, the ASFV genome was detected in the food of a traveler from Vietnam to Taiwan in February 2019, raising suspicion that ASFV was already widespread in the country before the official report (87). In 2020, the outbreak spread further, reaching 771 cases across Southeast Asia, with more than 550 outbreaks reported in the Philippines alone. In 2021, 87 new ASF outbreaks were reported in Malaysia, of which about 40% originated from wild pigs. In the same year, Thailand officially reported an ASF outbreak, and the following year 117 outbreaks were reported. The most recently infected country in this subregion is Singapore, where the disease was reported in wild boars in February 2023.

The virus strains isolated in Southeast Asian countries (Vietnam, Indonesia, and Malaysia) are highly homologous to each other and genetically similar to the genotype II ASFV isolated in China (88–90). Indeed, there are frequent reports of illegal cross-border movement of animals and meat products between China and Vietnam (11, 90, 91). Genetic analyses of ASFV isolates from domestic pigs in northern Vietnam have shown the continuous introduction of Chinese ASFV strains via illegal trade (92, 93), further highlighting that illegally attenuated vaccine strains of ASFV recently discovered in China have already spread to neighboring countries (90, 94). As many Southeast Asian countries share land borders, pig traders move across borders, and evidence of ASF infection has been found in brought-in pigs and pork products at various locations (95, 96). From the perspective of the EMPRES-i database, Vietnam and the Philippines have continuously reported numerous outbreaks since the early stages of the epidemic, with the total number of notifications exceeding 1,000 in both countries. In Vietnam, the disease had spread to all provinces within 5 months of the first ASF confirmation, killing nearly 6 million pigs, which is more than 20% of the country’s pig production (97). In the Philippines, at least 300,000 pigs have been culled (98). These massive ASF epidemics not only affected farmers but also caused pork prices to soar, which greatly affected the livelihoods of consumers. The number of notifications in Indonesia recorded in EMPRES-i is small (43 notifications as of the end of June 2023), however, the outbreak was confirmed in 10 of the 34 swine industry provinces, killing over 3.5 million pigs (99).

### 4.2. Pig industry and wild boar distribution

#### 4.2.1. Pig industry

Pigs play an important role in the lives of rural and peri-urban populations in Southeast Asia, and pork is the preferred meat in most countries. Types of pig production vary, ranging between small family units of backyard scavenging pigs, small to medium-sized semi-commercial units, and large intensive units. Like other Asian countries, the predominant practice is small-scale backyard farms with no or limited biosecurity, which are the most vulnerable to disease risks (14, 96, 100). The role of the pig industry varies among countries (101). Vietnam has a large domestic demand for pork, constituting 60% of all livestock production and raising the largest number of pigs in Southeast Asia at 30 million heads (97). In general, pig herds are very small, with about 49% being raised on small pig farms or backyard family farming units (102). Compared with the north, the south has more intensive and larger production systems (101, 103).

The overall pig stock in the Philippines was estimated at 9.49–12.7 million heads (104, 105). Of the total pig production, 70.6% are raised on private farms, while the remaining 29.4% belong to commercial farms. There are large pig farms in some areas of the country, however, backyard pig farming still accounts for 65%–83% of the total in rural areas. The average number of pigs per backyard holding is extremely limited, with many backyard families keeping one or two pigs fed on crops (104). In Thailand, the majority of pig-farming households are small-scale farmers (93.51%), with 9.5 million pigs (106). In recent years, the country has been shifting toward an intensive production system and is likely to form part of an integrated supply chain (107). Some of the live pigs and pork is exported to neighboring countries but it is primarily for domestic consumption. Large commercial pig farms are concentrated in peri-urban areas, while smaller pig producers are often found in rural and remote areas (107).

To meet the growing demand for pork in Cambodia and Laos, imports of live pigs and pork from neighboring countries such as Thailand, Vietnam, and China, along with the domestic pig farming industry, are increasing. In Laos, as of 2020, approximately 4.3 million pigs are allocated to about 580 commercial pig farms (108); in Cambodia, around 70% of pork is supplied by small-scale farmers (109). This trend of increasing pork demand is the same in Myanmar, with about 19.19 million pigs being raised in the country as of 2020 (110). Most pig farmers are small-scale farmers practicing free-range or backyard animal husbandry, and every household in the village raises at least one pig. This is not only for residual waste disposal but also for additional income (111).

About 8.9 million pigs were distributed in 34 of the 38 provinces of Indonesia before the ASF outbreak, with approximately 80% of pigs being produced by small-scale farmers holding less than 20 sows (88, 101). Although production is for domestic consumption, the pork-consuming population constitutes just 13% of the total due to the large Muslim population (101). Likewise, in Malaysia, having a large Muslim population, an estimated 1.7 million pigs are raised on 614 farms as of 2020, mainly for the country’s ethnic Chinese population. The majority of pig farms in the Malay Peninsula still operate on an open-house system (112).

The pig farming situation in Timor-Leste differs slightly from other Asian countries, where almost the entire domestic pig herd is held by small-scale farmers (113). Approximately 450,000 pigs are kept in the country in both urban and rural areas, with an average of less than three pigs per household, distributed to approximately 70% of the total population (114). As in rural areas of other Asian countries, livestock tend to be perceived as part of the family or property, rather than just for commercial purposes (115). Singapore has relied on imports since pig farming was discontinued in the early 1990s (116). ASFV was detected in carcasses at slaughterhouses in Singapore after live pigs were imported from Indonesia in April 2023 (117).

#### 4.2.2. Wild boar distribution

The Eurasian wild boar (*Sus scrofa*) is an endemic species in Southeast Asia and is widespread across forested areas (118–120). Their average density remains unknown, however, high densities of 30–40 animals/km^2^ have been recorded in some areas, e.g., conservation areas in Indonesia, Malaysia, and Singapore (121). Accordingly, the potential risk of ASF infection in wild boars has been discussed (72, 122, 123). Surveys conducted in Vietnam, Laos, and Cambodia found extensive overlap between wild boar habitats and domestic pig sites around villages adjacent to forests in these countries (124). Numerous interactions between wild boars and domestic pigs have been documented owing to the common practice of free-ranging domestic pigs (96). This creates a high-risk interface for virus transmission between these groups (121, 124). While the presence of ASF in wild boars in Laos and Vietnam was confirmed, the role of wild boars in the transmission cycle of ASFV in this region was concluded to be uncertain (124). Besides the endemic wild boar (*Sus scrofa*), the disease is feared to have a potentially serious impact on 11 endemic wild pig species in Southeast Asia (125). Despite this, ASF notifications have been limited to incidental reports of mortality events in Bornean bearded pigs (*Sus barbatus*), wild boars (*Sus scrofa*) in Laos and Vietnam, and warty pigs (*Sus cebifrons*) in the Philippines (124, 126, 127).

### 4.3. Risk factors and control measures

The Southeast Asian swine industry faces several major problems: low biosecurity swine production systems dominated by small farmers; complex, multistage, integrated production systems; illicit transportation of pigs and/or pork products with insufficient monitoring caused by price differentials and social factors; and cross-border disease spread through long and porous borders (128).

More than 90% of outbreaks in Vietnam’s early epidemics occurred on small and medium-sized farms with poor biosecurity, raising challenges for ASF prevention and control (97). As in China, the small farm sector is declining but may take time to be fully replaced by modern commercial farms (97). In these areas, people often cannot properly dispose of infected animals and dump the carcasses in rivers or roadside shrubs after slaughter, causing the disease to spread even further (102, 122, 129, 130). This can be partially explained by the limited capacity of veterinary services to deal with epidemics at the municipal level. Poor public veterinary services in the field lead to diseases not being properly diagnosed and contribute to their expansion (131). Similar practices due to the lack of biosecurity knowledge as well as the limitations of veterinary services have been observed throughout Asia (32, 96, 106, 132, 133). While these risk factors emphasize the importance of implementing strict biosecurity measures on small farms, the absence of stringent surveillance entails the risk of worsening the epidemic situation due to increased trade and consumption of infected animals (134).

Financial compensation after a disease outbreak on a farm is known to have a significant impact on farmers’ behavioral patterns, including their motivation for reporting (135). Full compensation may lead to lax preventive behavior, while inadequate compensation would encourage illegal trade and underreporting of cases by farmers (67). This is a common concern for many Asian countries, where financial compensation is often inadequate (63, 64), infected meat is sent to markets and/or restaurants to hide the outbreak, and the food waste could reach another pig farm as leftovers due to swill-feeding practices (136). A study conducted in Vietnam indicated that ASF surveillance data may have been underreported due to the lack of awareness, animal health professionals, and laboratory facilities in rural areas. In particular, farmers were reluctant to report to the authorities because of low compensation rates and complicated, lengthy administrative procedures (122). An attempt to sell suspect pigs was also observed, even at a lower price before ASF was confirmed, rather than waiting for longer to obtain higher compensation (97). Note that these are problems on a regional scale, not on a farm unit basis. In the Philippines, local communities hid sick pigs to avoid culling their pigs (132).

The pork food system in Vietnam is becoming more complex and large-scale through rapid economic development (137, 138). Also, the predominance of fresh meat being sold via the wet market poses a major vulnerability in the pork marketing system from the perspective of risk management for disease spread (67, 139). The pig trade depends on market demand and price differentials; traditionally, town traders, such as slaughterhouse operators and market sellers, go to the villages to purchase pigs to supply local demand. Improvements in road infrastructures have facilitated long-distance trade from rural producers to large cities and even to foreign markets. However, the scarcity of effective tracing systems in most areas makes it hard to monitor pig movements, and unregulated movements are common (128). As a result, illegal cross-border transportation frequently occurs. In a spatial risk assessment study of ASF introduction in Thailand, distance from the border was identified as one of the highest-priority risk factors. Consequently, several ASF outbreaks are now reported in many of these land-bordering areas (140). All frequently used distribution routes, not just road transportation networks, require attention. In the island nation of Indonesia, ports have been identified as a contributing factor to ASF outbreaks because of the daily marine transportation of pigs (141). Similarly, food waste from overseas vessels is an important virus transmission pathway. A study conducted at an Indonesian port found an ASFV prevalence of 8.69% in food waste brought in by ships from China and the Philippines (141).

Southeast Asian countries also have seasonal patterns in ASF outbreaks, as in China and South Korea. The increased movement of people and/or animals during the Vietnamese New Year may have contributed significantly to the nationwide spread of the virus (122). In the Philippines, environmental factors and social practices possibly contribute to a seasonal pattern in ASF outbreaks. The third quarter, coinciding with the beginning of the academic year, is a time when small farmers tend to sell their pigs to finance education, leading to the frequent movement of livestock and pork products throughout the country. Moreover, a significant increase in precipitation during the rainy season presumably leads to the dispersal of carcasses and environmental contamination, thus contributing to the higher frequency of ASF outbreaks (98, 132).

Border controls have been tightened in many countries to prevent the entry of pigs and pork products from ASF-infected areas. In addition, the application and proper management of biosecurity on pig farms together with rigorous and intensive monitoring of high-risk areas are recommended as important strategic steps to prevent ASF. In the Philippines, the government implemented various policies and public health strategies in response to the epidemic. A series of actions, called the 1-7-10 Protocol, established the application of zoning-based culling and active surveillance activities and testing. In 2021, the National African Swine Fever Prevention and Control Program (BABay ASF) was launched to prevent and control ASF via surveillance, monitoring, and repopulation efforts (98). The Vietnamese government endorsed the “National plan for the prevention and control of African swine fever for the period 2020–2025,” which defines the ASF management process from farm biosecurity adaptations to laboratory capacity development in July 2020 (142). The plan includes the application of partial culling due to the difficulty of applying this measure to all animals. This approach has the great advantage of significantly reducing livestock losses, nevertheless, it can increase the risk of a prolonged disease epidemic period unless high biosecurity levels can be maintained (143, 144). Furthermore, infected farms tend to retain and raise recovered pigs to minimize losses and shorten the time to reintroduction. Recovered pigs can progress to chronic infections and thus are a potential source of infection, contributing to the current endemic situation in Vietnam (145).

There is scant information describing the role of wild boars in ASF transmission in Southeast Asia, however, their presence throughout the region suggests the high possibility of spreading and sustaining the ASFV (127). The fewer ASF notifications in wild boars in Southeast Asia are inexplicable given their high densities, gregarious social behavior, opportunities for contact with domestic pigs, and the landscapes they occupy (146). Given reports of contact between free-ranging pigs and wild boars in rural areas, besides the lack of adequate surveillance systems, they may play a role in the spread and maintenance of the disease (96). Indeed, a study that spatially quantified the predicted risk of ASFV infection in wild boars across Asia identified Southeast Asia as concentrating the highest risk areas (72).

## 5. ASF subregional update in South Asia

### 5.1. ASF epidemic status

**Figure 7:**
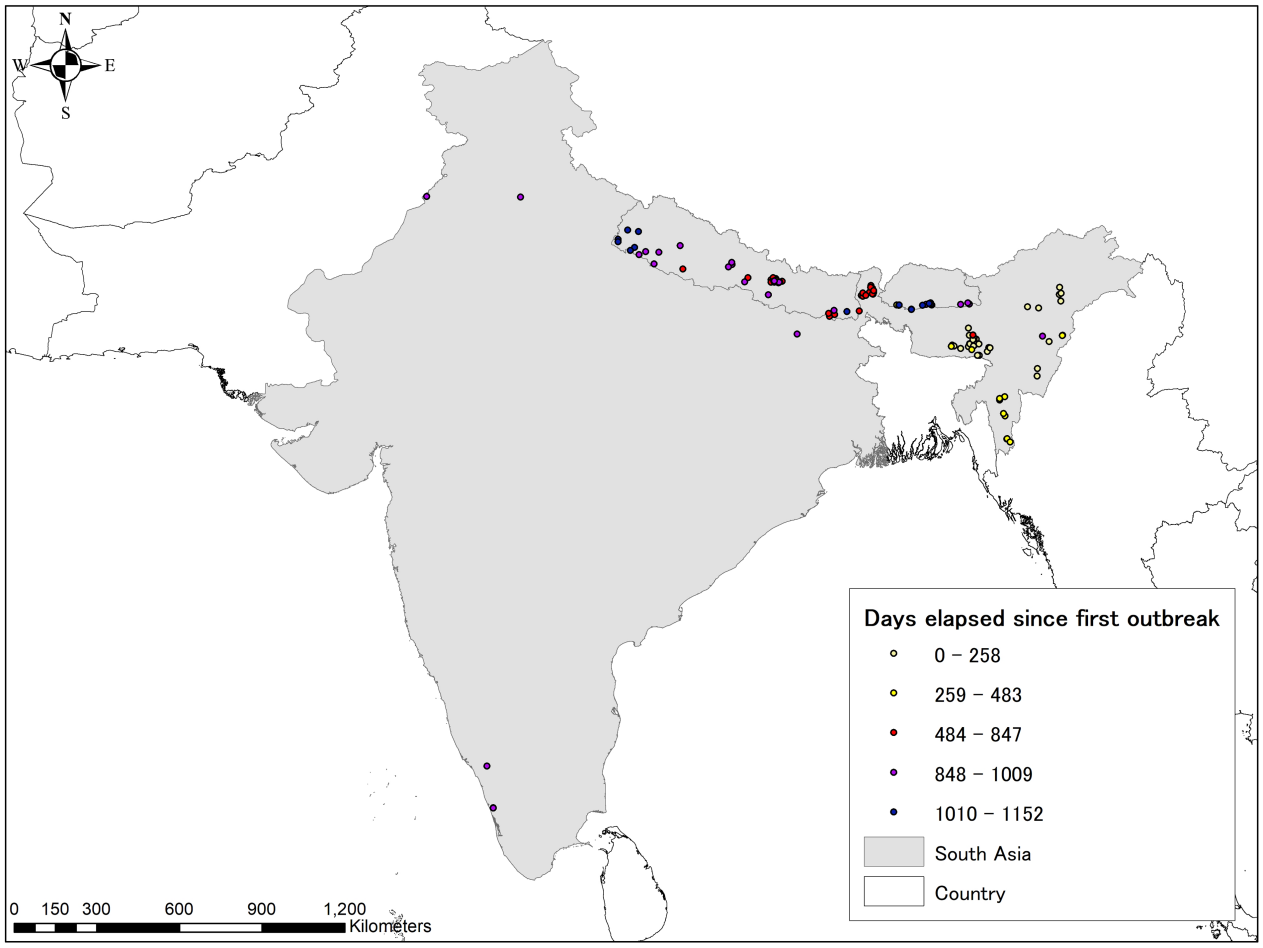
ASF evolution in South Asia as of June 30, 2023, based on the EMPRES-i database. Lighter colors indicate earlier stages, darker colors indicate more recent occurrences in this region. The map was depicted using ArcGIS 10.8.1 (ESRI, Redlands, CA, United States).

South Asia is a relatively new region for the emergence of ASF, with the disease confirmed in three countries to date (India, Bhutan, and Nepal). Compared with other Asian regions with outbreaks spreading across the entire region, the spatial distribution of ASF is centered in the northeast area, which appears to be gradually spreading westward (Figure 7). The first ASF infection was confirmed in India in January 2020. Abnormal swine mortality was reported in Assam and Arunachal Pradesh in early January 2020 and later diagnosed as ASF positive (147). The virus strains isolated were 100% identical in nucleotide sequence to ASFV in Asia and Europe, including China, South Korea, Vietnam, Georgia, and Hungary (148). The following year, Bhutan became infected in May, and Nepal confirmed its first case of ASF in March 2022. The precise decrease in pig numbers due to ASF is unknown, but approximately 54,000 pigs died by July 2021 in India (149). Throughout the region, 130 notifications have been reported to FAOEMPRES-i to date, all from domestic pigs except one case from wild boar. In 2022, 63 notifications, the highest number to date, were recorded, owing to the large number of outbreaks observed in Nepal, along with ongoing outbreaks in India. As of the end of June 2023, outbreaks continue to be reported from various areas.

### 5.2. Pig industry and wild boar distribution

#### 5.2.1. Pig industry

Approximately 9 million pigs are raised in India, 45% of which are in the northeastern states (150). The northeastern region has the largest pig population, followed by eastern, southern, central, northern, and some western regions of India (147). About 90% of pigs are raised by resource-poor smallholder farmers (149), and pig farming is of great importance for the livelihoods of the rural poor, especially in these states (148). Swill feeding is common, with pigs roaming freely for food in both rural and urban areas. Among small farmers, traders usually travel between villages to collect pigs and bring them to livestock markets and slaughterhouses (151). Commercial pig farms with large-scale pig production in India are scarce and are mostly found in peri-urban areas (152).

The demand for the pork industry in Nepal has increased significantly in recent years, with the number of pigs increasing from 1.1 million in 2011 to 1.6 million in 2021 (153). Although there are some modern pig farms, the majority of these are dominated by small-scale farmers (154). In common with pig production in India, most pigs are raised by scavenging activities utilizing food waste (155). Many of these pigs are slaughtered on their farms due to the lack of slaughterhouses (156).

#### 5.2.2. Wild boar distribution

Information on wild boar populations and distribution throughout South Asia is not available. However, the Indian crested boar (*Sus scrofa cristatus*) is found in most protected wildlife areas and is widely distributed in India, Sri Lanka, Nepal, Thailand, and Myanmar (157). The northeastern states of India, especially those with forest cover exceeding 65% and large wild boar populations, are considered a major threat to the spread of ASF infection (147, 149, 158).

### 5.3. Risk factors and control measures

Many rural farmers lack general knowledge about infectious diseases and often fail to report infections. Subsequently, the risk of disease spread is high when animals from uncertain sources are purchased. In most rural and remote areas, pigs are slaughtered on home grounds or in open meat markets in the absence of organized abattoirs, and the run-off derived from these slaughterhouses is directly accessible to animals. Free-range pig production, the movement of virus-contaminated pigs, and lack of basic biosecurity measures are major risk factors in India, as in other Asian countries (147). In Nepal, the first ASF outbreaks in various swine production areas in the Kathmandu Valley were suspected to be caused by swill feeding (159).

Many of the Northeastern states of India share borders with Tibet, China, Myanmar, and Bangladesh, and there are no restrictions on the movement of people or goods, thus posing a continuous risk of ASF introduction into the country (150, 160). This is shared with other countries, and Nepal also suggests a risk of pigs entering illegally across the border. Additionally, there is a continuous transmission risk of ASF to wild boars via forest routes adjacent to India-Nepal National Parks and Reserves (154).

No official cases of ASF in wild boars have yet been reported in India, nonetheless, a wild boar carcass found in a northeastern state was positive for ASF (161). It is more likely that the disease originated from infected domestic pigs rather than spreading among wild boars. As confirmed in other countries, disposal of infected carcasses in rivers during the early stages of the epidemic may have caused further spread of the disease (147). ASF outbreaks have been reported around the Brahmaputra River, a tributary of which flows through national parks and wildlife sanctuaries in northeastern India. Most of the densely distributed domestic pigs in this area are backyard farms with inadequate biosecurity measures and are a major threat to the wild boars that abound in this area (147). In addition, several states in the northeastern region are prone to flooding, raising concerns about the spread of ASF associated with animal movements (149). It is hypothesized that the early ASF outbreaks in India involved wild boars (direct transmission among wild boars, indirect transmission via their habitat, and contact between wild boars and domestic pigs) and the subsequent domestic transmission cycle involved disease transmission among domestic pigs via contaminated pig products/fomites (150).

There is limited research on soft ticks, particularly *Ornithodoros* species, in the region; their geographic distribution is yet to be defined. Moreover, there is no official evidence of the involvement of *Ornithodoros* species in the current ASF outbreak in northeastern India. However, studies associated with soft tick distribution modeling are considered very important for disease prevention (147).

## 6. Discussion

**Figure 8:**
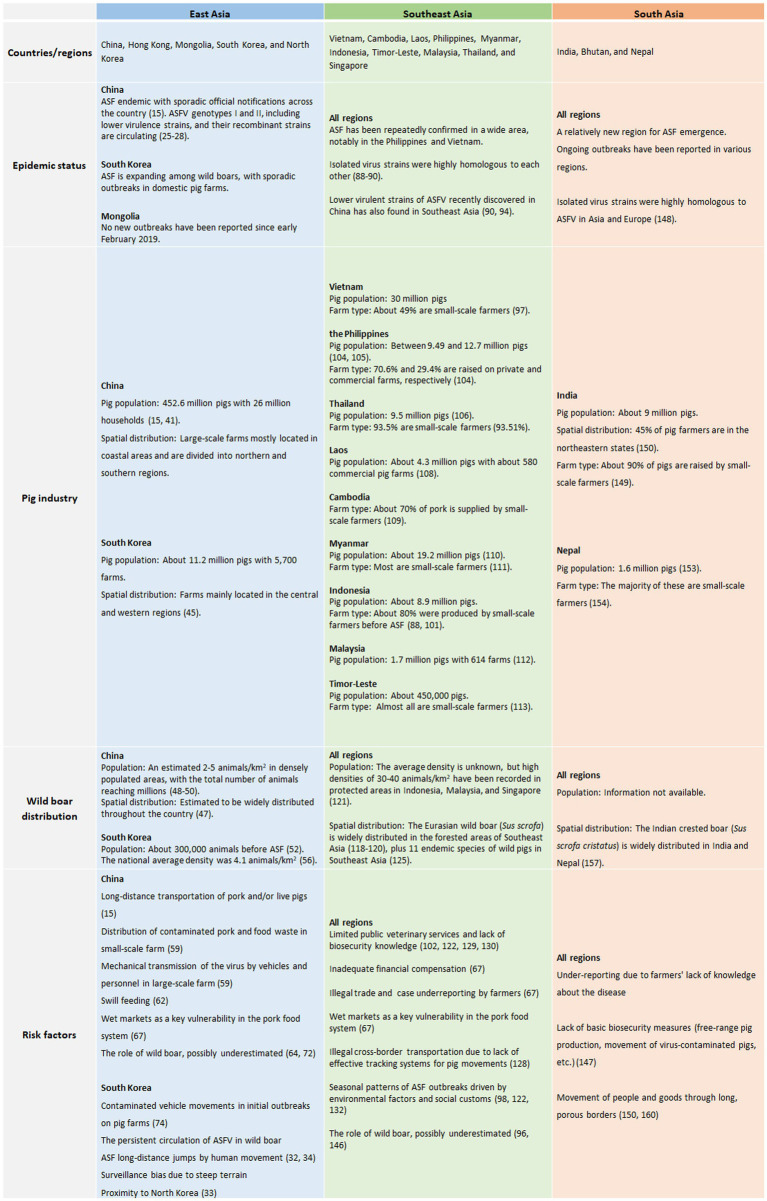
Brief summary of the epidemiological situation and relevant information for each region presented in this study.

The epidemiological status and related information for each of the regions described above are briefly summarized in Figure 8. The ASF epidemic situation in Asia has become more complex and disease control more challenging. Apart from Mongolia, where all ASF events have been resolved, the disease is still widely distributed throughout affected Asian countries. As the world’s top pork-producing countries include China and other Asian countries such as Vietnam, South Korea, Japan, and the Philippines, more serious consequences for the entire global swine industry can be anticipated. Small-scale farmers with low biosecurity levels have traditionally played an important role in pork production in most Asian countries, implying that ASF management will be challenging. Note that this is not a problem exclusive to Asia, it is observed worldwide.

The information available regarding wild boars is limited, mostly sourced from South Korea, where wild boars play a major role in the expansion of ASF. However, this does not imply that the ASF risk of wild boar should be neglected in the other countries. Wild boars are abundant throughout Asia, and cases have been officially reported in half of the 18 ASF-affected countries/regions. In many countries, limited resources are allocated to wildlife surveillance (162), and, therefore, the potential underestimation of the wildlife epidemiological situation should also be fully considered (121, 123). Risk factors and their priorities differ among these countries, as this review has shown. ASF management strategies should aim to accommodate differences in swine husbandry, wild boar distribution, priority risk, culture, and social values across regions.

In this review, we have summarized the ASF outbreak situation in Asia based on officially reported information. However, the number of notifications does not always accurately reflect the epidemic status (162). For example, if a disease is endemic in a country’s territory, the WOAH standard allows these diseases to be reported in a six-monthly report (163). Also, each country has its own epidemiological unit for disease reporting, thus caution is required when interpreting simply by comparing report numbers. As a result, the number of disease notifications differs between WAHIS, EMPRES-i, and the country’s own databases, as queried by some literature (6, 7, 80, 98). Such discrepancies should always be considered, along with underreporting at the point of data collection. As small and medium-sized farms (<500 head) account for 99% of the swine industry in China, a complete and accurate picture of the number of slaughters and deaths on these farms is challenging to obtain (164). As mentioned above, backyard farms and small farms are the norm in affected Asian countries, hence this concern is likely to be common to most countries. One of the feared possibilities is that the disease becomes endemic, with periodic outbreaks affecting the food system (47). In some countries, the number of outbreaks has already subsided, with only sporadic reports from various locations; however, it has not been determined whether this is due to the data gap or reflects the actual situation. This review is based on publicly accessible information and published literature, which biases the amount of information by region. Paradoxically, this underscores the need for further research.

Before 2018, ASF was mainly distributed in Africa and Europe. The current epidemic status and the significance of the swine industry indicate that Asia and Europe are most likely to be the main players in the ASF epidemic for a time to come. The two regions are closely linked historically and geographically and have much in common. As the ASF expansion in Europe has influenced the emergence of ASF in Asia, the Asian ASF epidemic is surely a new concern for Europe, as well as the rest of the world. ASF control remains a top priority for the WOAH and FAO, hence initiatives are underway within the Global Framework for the Progressive Control of Transboundary Animal Diseases (GF-TADs) to implement risk-based control strategies on a regional scale. This includes technical assistance to Asian countries for ASF diagnosis and epidemiological interpretation of the situation (165, 166). Much has been learned in Europe over the past 16 years, yet not enough to contain the disease. In Asia, where this disease is spreading at an unprecedented rate, the importance of cooperation and collaboration between countries is emphasized, along with greater efforts for disease control (167). There is much to be learned from this experience to prevent another disaster.

## Author contributions

SI: Conceptualization, Writing – original draft, Writing – review & editing. NK: Conceptualization, Writing – review & editing. JB: Writing – review & editing. CA-V: Writing – review & editing. JS-V: Supervision, Validation, Writing – review & editing.

## Funding

The author(s) declare financial support was received for the research, authorship, and/or publication of this article. This research was funded by European Project H2020 VACDIVA—A Safe DIVA vaccine for African swine fever control and eradication, grant agreement no. 862874.

## Conflict of interest

The authors declare that the research was conducted in the absence of any commercial or financial relationships that could be construed as a potential conflict of interest.

## Publisher’s note

All claims expressed in this article are solely those of the authors and do not necessarily represent those of their affiliated organizations, or those of the publisher, the editors and the reviewers. Any product that may be evaluated in this article, or claim that may be made by its manufacturer, is not guaranteed or endorsed by the publisher.
